# *Notes from the Field:* Effects of the COVID-19 Response on Tuberculosis Prevention and Control Efforts — United States, March–April 2020

**DOI:** 10.15585/mmwr.mm6929a4

**Published:** 2020-07-24

**Authors:** Ann M. Cronin, Shanica Railey, Diana Fortune, Donna Hope Wegener, Justin B. Davis

**Affiliations:** ^1^Division of Tuberculosis Elimination, National Center for HIVAIDS, Viral Hepatitis, STD, and TB Prevention, CDC; ^2^The National TB Controllers Association, Smyrna, Georgia.

CDC’s Division of Tuberculosis Elimination (DTBE) funds 61 state, local, and territorial tuberculosis programs in the United States through the TB Elimination and Laboratory cooperative agreement. Recipients report data to CDC on indicators that measure progress toward TB elimination and performance of essential TB program activities. After the first U.S. case of coronavirus disease 2019 (COVID-19) was reported on January 20, 2020 (*1*), CDC project officers were informed by these grantees that program personnel (including those positions funded through the CDC cooperative agreement and state or local budgets) would be deployed for their jurisdictions’ COVID-19 response.

In April 2020, as part of routine monitoring, CDC project officers communicated with 50 of the 61 (82%) grantees to estimate the effect of COVID-19 deployments on essential TB activities. Eleven (18%) programs were not reached because of deployments among project officers and recipients. CDC project officers characterized the effect as 1) no impact (no changes in staffing assignments or TB program activities), 2) partial impact (<50% of personnel time dedicated to COVID-19 response or some changes made to program activity, but activity still being performed), or 3) high impact (50%–100% of personnel time dedicated to COVID-19 response or major changes made to program activity or activity not being performed at the time of the program’s response) (Table).

**TABLE:** Effect of COVID-19 response on CDC tuberculosis (TB) elimination and laboratory program performance indicators, by level of impact — 50 U.S. jurisdictions,* April 2020

Performance indicator	No. (%)			
	No impact^†^	Partial impact^§^	High impact^¶^	Partial^§^ or high^¶^ impact
Program staffing for cooperative agreement and fiscal management	18 (36)	16 (32)	15 (30)	**31 (62)**
Program staffing for clinical consultation or clinic service delivery	17 (34)	21 (42)	12 (24)	**33 (66)**
Program staffing for outreach and field services (e.g., directly observed therapy or contact investigations)	16 (32)	16 (32)	14 (28)	**30 (60)**
Program staffing for surveillance and case reporting	14 (28)	24 (48)	12 (24)	**36 (72)**
Program staffing for training and program evaluation	15 (30)	13 (26)	21 (42)	**34 (68)**
Diagnosis and treatment of persons with TB disease	23 (46)	22 (44)	4 (8)	**26 (52)**
Diagnosis and treatment of persons with LTBI	15 (30)	25 (50)	9 (18)	**34 (68)**
Contact investigations for infectious TB cases	17 (34)	23 (46)	9 (18)	**32 (64)**
Targeted testing and treatment of LTBI among populations at risk	12 (24)	22 (44)	15 (30)	**37 (74)**
Case reporting and other surveillance activities (e.g., genotype or cluster monitoring and data analysis)	20 (40)	24 (48)	5 (10)	**29 (58)**
Program evaluation activities (e.g., cohort review)	10 (20)	20 (40)	17 (34)	**37 (74)**
Education and training activities	2 (4)	22 (44)	25 (50)	**47 (94)**

**Abbreviations:** COVID-19 = coronavirus disease 2019; LTBI = latent TB infection.

* Reported by 50 of 61 CDC-funded TB program recipients to CDC project officers. Number and row percent might total <50 (100%) as a result of missing responses. Eleven programs could not be reached because of deployments among the eight CDC project officers or in the TB programs.

^†^ No changes in staffing assignments or TB program activities.

^§^ <50% of personnel time dedicated to COVID-19 response or some changes made to program activity, but activity is still being performed.

**^¶^** 50%–100% of personnel time dedicated to COVID-19 response or major changes made to program activity or activity was not being performed at the time of the program’s response.

Among the 50 programs, 60%–72% were experiencing partial or high impact on staffing capacity for 1) cooperative agreement and fiscal management, 2) clinical consultation or clinic service delivery, 3) outreach and field services (e.g., contact tracing and directly observed therapy), 4) surveillance and case reporting, and 5) training and program evaluation.

Changes in staffing capacity were assessed separately from changes in essential activities. For example, if staffing capacity had been reduced, nondeployed staff members could still have assumed additional, high-priority duties, such as ensuring patient care.

Partial or high impact on indicators measuring essential TB control activities was reported by 52% of jurisdictions for diagnosis and treatment of persons with TB disease, 68% for diagnosis and treatment of persons with latent TB infection, 64% for contact investigations for infectious TB, 74% for targeted testing and treatment of latent TB infection among populations at risk, and 58% for case reporting and other surveillance activities (genotype or cluster monitoring and data analysis). In addition, 74% of the TB programs reported reduced program evaluation, and 94% reported reduced education and training efforts.

The National TB Controllers Association (NTCA), which represents all state, local, and territorial programs, observed similar effects. NTCA convenes monthly webinars for members to discuss emerging problems and share best practices. By March, webinar participation was declining because of deployments. To obtain moment-in-time impressions of how the response was affecting TB activities, NTCA queried participants using a series of real-time text questions and tallied responses to each question. In the March 18 and April 9, 2020, webinars, >90% of 43 (March) and 38 (April) responses indicated that TB programs had deployed personnel to their jurisdictions’ COVID-19 response. TB program personnel possess skills that health departments needed for the response. For example, among 72 responses in April, 26% were providing expertise in contact tracing, 21% in infection control, 17% in clinical care and treatment, and 14% in monitoring patients in home isolation.

Responses to polling questions indicated that capacity for essential TB activities declined between March and April. For example, during the April webinar, the percentage of responses regarding less time for interviewing patients doubled over responses to the same question in March (22% of 115 responses in April, compared with 10% of 110 responses in March), and 15% indicated challenges in obtaining TB medications, up from 7% in March. Transfer of TB resources for COVID-19 use (including personal protective equipment, housing, hospital beds, and isolation rooms) was indicated by 12% of responses in April, up from 7% in March.

These observations suggest that the COVID-19 response is diverting resources from essential TB elimination activities. Effects of reduced capacity on outcomes (e.g., increases in TB incidence or lower completion of treatment rates) will become clearer after provisional surveillance data, including number of U.S. TB cases reported during 2020, are published in early 2021. CDC is monitoring state capacity for reporting TB cases and will document gaps in reporting associated with the COVID-19 response. However, signals of reduced capacity are concerning. Incomplete contact investigations and delays in diagnosis of TB disease are associated with outbreaks of TB disease (*2*), and sustained weakening of TB programs was recognized as a cause of the TB resurgence in the late 1980s and early 1990s (*3*).

The COVID-19 response has affected multiple sectors of public health, recommended preventive screening, and clinical care. The United States will need to address the backlog of population health services that have been delayed or not done while public health resources are focused on COVID-19. The U.S. domestic TB elimination program is one example. If essential TB program activities are not sustained, gains made in reducing U.S. TB cases will be at risk. CDC has published guidance regarding non–COVID-19 public health activities that require physical interaction with clients.* CDC will support grantees by providing technical assistance or outbreak response, when requested. NTCA will continue to communicate with members and share best practices for averting a resurgence of TB.

## Footnotes

* https://www.cdc.gov/coronavirus/2019-ncov/hcp/non-covid-19-client-interaction.html.
